# Iron Deficiency Anemia Due to High-dose Turmeric

**DOI:** 10.7759/cureus.3858

**Published:** 2019-01-09

**Authors:** Thomas J Smith, Bimal H Ashar

**Affiliations:** 1 Oncology, Johns Hopkins Sidney Kimmel Comprehensive Cancer Center, Baltimore, USA; 2 Internal Medicine, Johns Hopkins Medical Institution, Baltimore, USA

**Keywords:** turmeric, curcumin, iron deficiency anemia

## Abstract

Turmeric is increasingly studied as an anti-inflammatory and anti-neoplastic agent. It binds to ferric iron in the gut and causes iron deficiency in mice. We report here a possible case of iron deficiency anemia in a human taking turmeric.

A 66-year-old physician treated himself for an osteoarthritis flare after steroids with six turmeric extract capsules (538 mg) daily, to help with inflammation. During this time, his hemoglobin never rose above 12 and his iron and ferritin levels were consistent with iron deficiency. Upper and lower endoscopy and Hemoccult™ studies were negative. Two weeks after stopping the turmeric and continuing his usual iron supplement, his hemoglobin had returned to normal, with normalizing iron studies. Turmeric was associated with significant iron deficiency anemia, consistent with the binding of available iron in the gut and the prevention of absorption. This resolved after the turmeric was stopped, consistent with animal studies.

This may be the first case of documented iron deficiency anemia in people due to turmeric supplements. Given the widespread use of turmeric and curcumin supplements across many illnesses, further attention is warranted.

## Introduction

Turmeric, the natural source of curcumin, is under intense investigation as an anti-neoplastic and anti-inflammatory agent in a multitude of conditions [1-2]. It is known to bind iron in the gut and can cause iron deficiency in mice [3]. Attempts to target the iron metabolic pathway with drugs such as curcumin are an exciting new way to prevent and treat cancer [4]. We report here the first potential case of iron deficiency anemia in a human, which was caused by turmeric.

## Case presentation

A 66-year-old physician was treated for a prostate-specific antigen recurrence of prostate cancer with radiation and six months of androgen depletion therapy with leuprolide (Eligard®, Tolmar Pharmaceuticals, Illinois, US) and bicalutamide (Casodex®, AstraZeneca Pharmaceuticals, Maryland, United States) starting October 11, 2017. He finished radiation on February 5, 2018. During this time, he developed significant myopathy with a decline in his marathon times, as reported in one other highly trained athlete [5]. He had pulmonary function tests (PFTs) done on July 5, 2018, showing forced expiratory volume (FEV1) at 61% of the normal, total lung capacity (TLC) at 65% of predicted, and diffusing capacity of the lungs for carbon monoxide (DLCO) at 75% of predicted. The workup for interstitial lung disease and neuromuscular causes of restrictive lung disease were negative. He was started on 40 mg of prednisone for a week, then 30 mg for three additional weeks to treat possible bronchiolitis. His muscle weakness worsened, so the prednisone was tapered to zero over three weeks beginning August 2, 2018. At the end of this taper, he developed substantial tendinitis, arthritis, and a small left knee effusion where he had existing chondrocalcinosis [6]. The rheumatologic workup was negative except for an increased sedimentation rate, up from eight to 29. To combat the near-crippling inflammation and arthritis, he began the intake of turmeric extract on September 13, 2018, with about six 538 mg turmeric extract capsules a day [7]. Doses up to 12 grams per day have been reported to be safe and well-tolerated [8].

**Figure 1:**
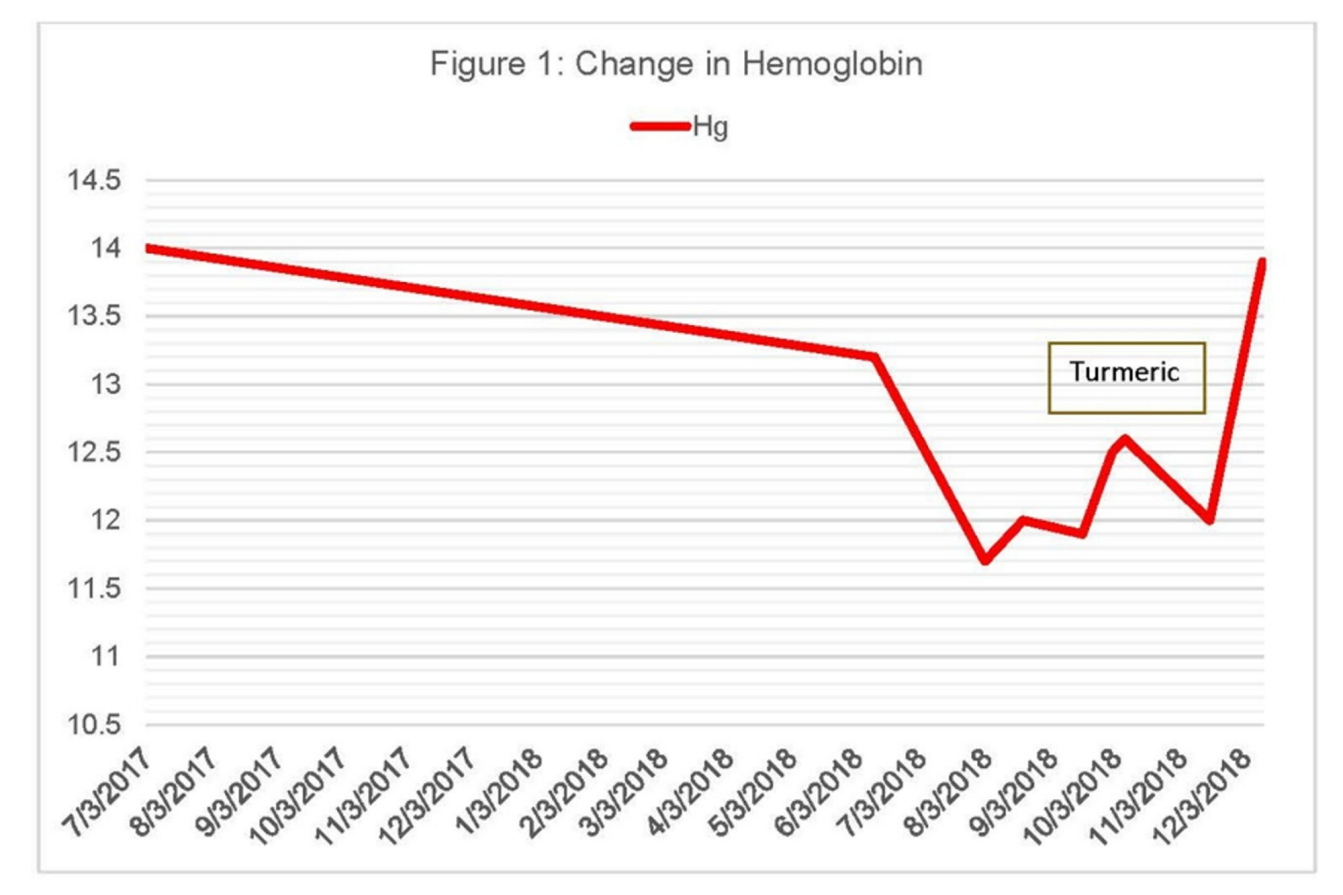
Change in Hemoglobin

**Figure 2:**
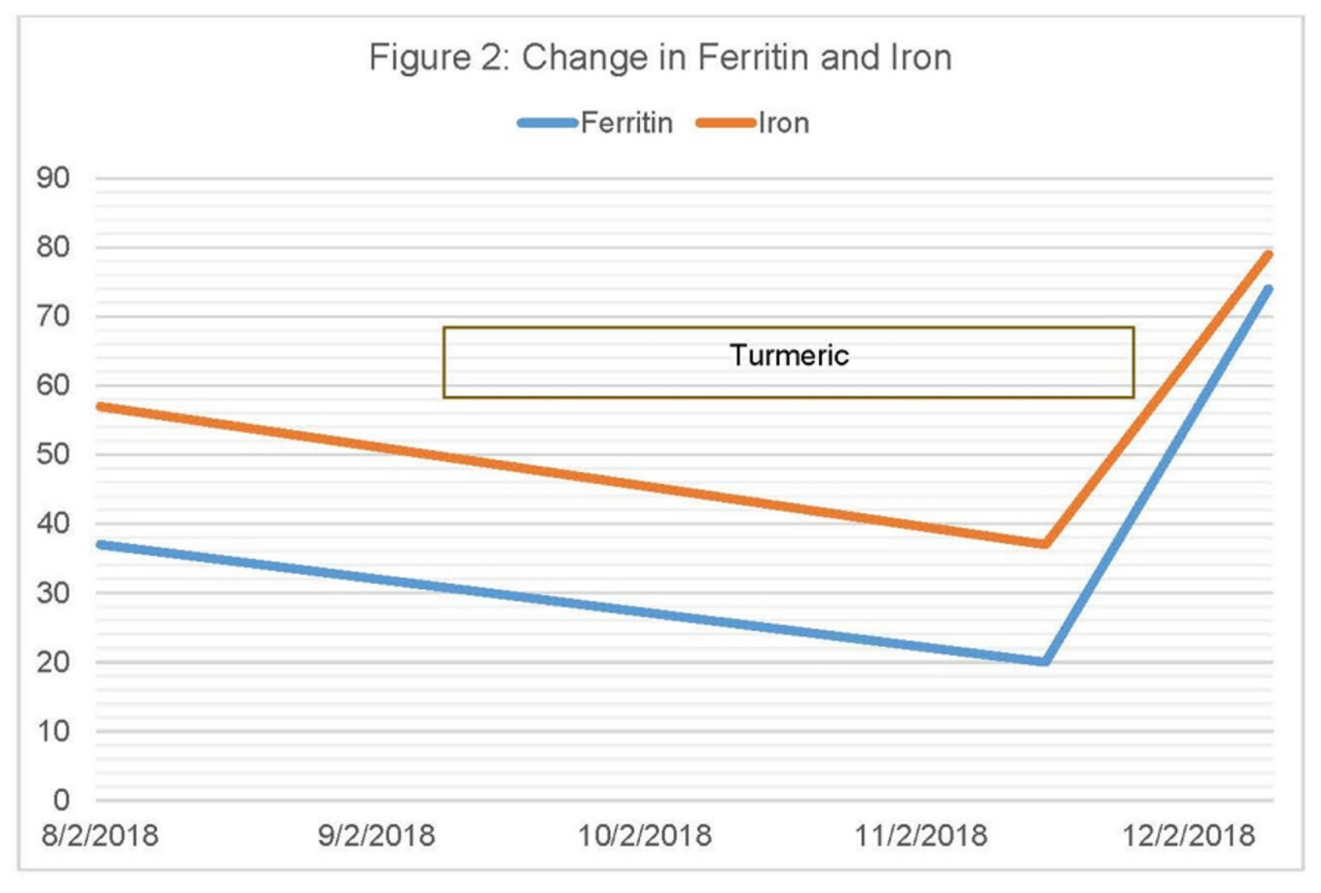
Change in Ferritin and Iron

Prior to starting radiation and androgen deprivation therapy, his hemoglobin was normal at 14.0 g/dL. He was an active red cell donor, having given over 100 units in the past 20 years, with low iron reserves [9] requiring iron supplements after prostatectomy in April 2016, when his hemoglobin fell to 9.8 g/dL. During and after radiation and androgen deprivation, his hemoglobin remained above 13.0 g/dL. During the period of respiratory dysfunction in July, his hemoglobin was found to be 11-12, so workup was begun. His ferritin and iron were both low, so he began iron supplementation with “Gentle Iron” (Nature's Bounty, New York, United States), containing 28 mg of elemental iron twice a day. Despite the supplements, his iron and ferritin continued to drop. Stool Hemoccult™ was negative. Upper and lower endoscopies in November were both completely normal. After the negative endoscopies, he reviewed the literature on a possible turmeric-related iron deficiency and stopped the turmeric. Within two weeks, his iron, ferritin, and hemoglobin were rising while taking the same iron supplement, with no changes in his medical regimen (Figures 1-2). His tendinitis and arthritis mildly worsened and then stabilized, and his lung function improved.

## Discussion

We report the first case of a possible human iron deficiency due to the absorption of iron in the gut due to turmeric. While causality cannot be readily determined, the patient’s hemoglobin, iron, and ferritin were all reduced after he started turmeric and returned to normal after he stopped it. No other cause of iron deficiency or blood loss was found on extensive evaluation.

Turmeric is among the spices known to inhibit iron absorption by 20%-90% in humans, reducing iron absorption in a dose-dependent manner [10]. The stoichiometric qualities of turmeric indicate it could bind nearly all absorbable iron and cause iron deficiency, and it does so in mice [3]. Curcumin, the active ingredient in turmeric, binds ferric iron (Fe3+) to form a ferric-curcumin complex that is dose-dependent and Fe3+ specific [11]. In mice, liver hepcidin and ferritin expression were strongly suppressed and iron concentrations in the liver and spleen were reduced by over 50% (3). Curcumin represses the synthesis of hepcidin, one of the peptides involved in iron balance, and has the potential to induce iron deficiency in the setting of prior subclinical iron deficiency [12].

iron deficiency associated with turmeric has been reported in the lay literature, with several patients describing anemia that responded to stopping turmeric (https://www.consumerlab.com/answers/does-turmeric-curcumin-reduce-iron-absorption-from-food/turmeric-curcumin-iron/; ConsumerLab.com; Accessed: January 8, 2019). At least one hemochromatosis website recommends turmeric for its benefit in arthritis and potential benefit in reducing excess body iron. (https://hemochromatosishelp.com/turmeric-benefit-for-hemochromatosis/; Accessed: January 8, 2019). While labs were not drawn to assess the effect of turmeric on anemia, the rapid recovery of hemoglobin from 12 to 13.9 g/dL in two weeks off turmeric, with no other changes in the medical regimen, suggest an association.

## Conclusions

Turmeric supplementation is increasingly common. Humans presenting with iron deficiency anemia should be queried about supplement use. The ability of turmeric to absorb intestinal iron may lead to it being useful in states of iron overload, such as hemochromatosis, or hemolytic anemias, such as sickle cell disease.
